# On Sleep and Insomnia

**Published:** 1865-08

**Authors:** 


On Sleep and Insomnia. By Wm. A. Hammond, M, D., of New York, 1865.

This book is in pamphlet form, and is divided into two parts.

Part first discusses the physiology of sleep. After giving a number of illustrations the author says: "The quantity of blood circulating through the brain during sleep is decidedly less than during wakefulness, and that sleep may be directly induced by arresting the flow of blood to the brain."

Part second speaks of the pathology and treatment of insomnia. In this the author records a number of very interesting and instructive cases with their treatment, in some of which the bromide of potassium administered in thirty grain doses was found to be beneficial.

The book is a re-print from the New York Medical Journal for May and June. To those interested in this variety of disease, this work will be found exceedingly valuable. K.

